# Design and Synthesis of Molecular Hybrids of Sophora Alkaloids and Cinnamic Acids as Potential Antitumor Agents

**DOI:** 10.3390/molecules25051168

**Published:** 2020-03-05

**Authors:** Hai Shang, Lingyu Li, Liyan Ma, Yu Tian, Hongmei Jia, Tao Zhang, Meng Yu, Zhongmei Zou

**Affiliations:** 1 Institute of Medicinal Plant Development, Chinese Academy of Medical Sciences and Peking Union Medical College, Beijing 100193, China; hshang@implad.ac.cn (H.S.); lyli@implad.ac.cn (L.L.); lyma@implad.ac.cn (L.M.); ytian@implad.ac.cn (Y.T.); hmjia@implad.ac.cn (H.J.); tzhang@implad.ac.cn (T.Z.); myu@implad.ac.cn (M.Y.); 2 State Key Laboratory of Bioactive Substance and Function of Natural Medicines, Institute of Materia Medica, Chinese Academy of Medical Sciences and Peking Union Medical College, Beijing 100050, China

**Keywords:** sophora alkaloid, hybrid, cinnamic acid, antitumor, apoptosis

## Abstract

Twenty-five sophora alkaloids-cinnamic acid hybrids (including matrine-cinnamic acid hybrids, sophoridine-cinnamic acid hybrids, and sophocarpine-cinnamic acid hybrids) were designed, synthesized, and evaluated in vitro against three human tumor cell lines (HeLa, HepG2 and A549) with cisplatin as a positive control. Some matrine-cinnamic acid and sophoridine-cinnamic acid compounds exhibited potent effect against all three cancer cell lines, such as compounds **5b**, **5e**, **5g**, and **6d**. The structure-activity relationship study of the synthesized compounds was also performed. Preliminary mechanistic studies indicated that compounds **5e** and **6d** could induce apoptosis in HepG2 cell line. Further, compounds **5e** and **6d** altered mitochondrial membrane potential and produced ROS leading to cell apoptosis of HepG2 cells. Overall, our findings suggested that these compounds may provide promising lead compounds for further development as antitumor agents by structural modification.

## 1. Introduction

**Figure 1:**
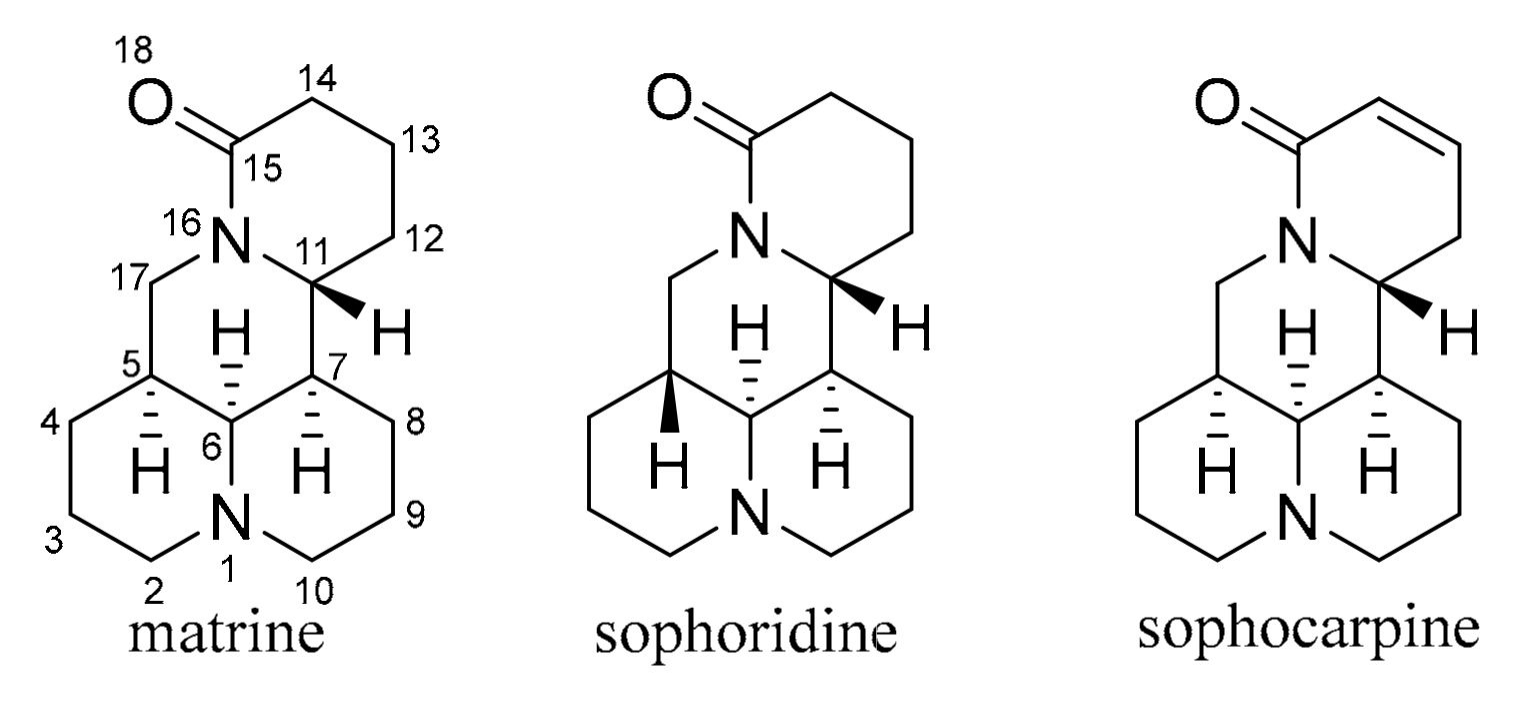
Structures of matrine, sophoridine, and sophocarpine.

Matrine, sophoridine, and sophocarpine (Figure 1) are the main extractable alkaloid components in the roots of *Sophora flavescens* Ait (Fabaceae), a common traditional chinese herb. They share the same core structure of quinolizidine and exhibit broad ranging bioactivity profiles that include antitumor [1,2,3,4], anti-inflammatory [5,6,7,8], anti-fibrosis [9], anti-viral [10,11,12], antimicrobial [13], and immunomodulatory effects [14]. It is noteworthy that their antitumor activity has attracted special attention [15,16,17,18,19,20]. Some studies have shown that matrine exerted antitumor effect through inhibiting the proliferation and inducing apoptosis of certain cancer cell lines [21,22,23,24]. Sophoridine, a 5*R* isomer of matrine, also has antitumor effect and was approved by the Chinese FDA in 2005 as an anticancer drug against malignant trophoblastic tumors [25]. However, the clinical applications of the sophora alkaloids remain limited because of their moderate antitumor activities and short half-life [9,26,27]. As a result, development of their derivatives aiming to improve their therapeutic efficacies is of great importance.

**Figure 2:**
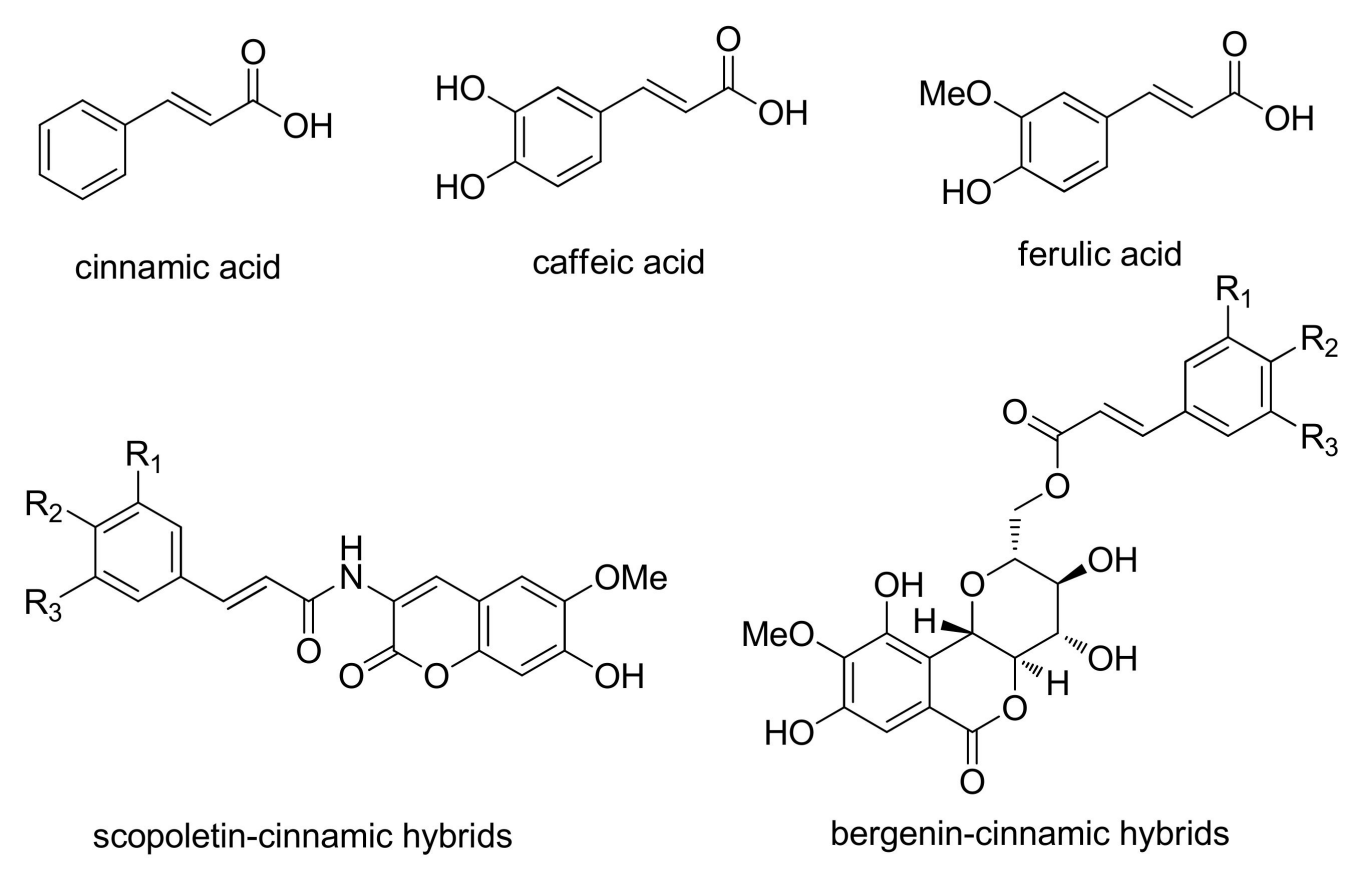
Structures of natural cinnamic acids and the hybrid derivatives of cinnamic acid.

Molecular hybridization is an attractive strategy in drug design by the combination of two active compounds or pharmacophoric units to produce a new hybrid compound with improved affinity and efficacy, when compared to the parent drugs [28]. Additionally, this strategy can result in compounds presenting modified selectivity profile, different and/or dual modes of action and reduced undesired side effects. Cinnamic acid and its analogues, such as caffeic acid and ferulic acid (Figure 2), are naturally occurring aromatic fatty acids which are widely presented in fruits, coffee, and wine [29]. They consist of a common phenyl ring substituted with an acrylic acid group and displayed a variety of biological activities including antitumor effect [30,31,32,33,34]. Owing to their unique structure and impressive biological activity, cinnamoyl moiety was often introduced into many natural and synthetic compounds in the design of antitumor drugs and the synthetic cinnamic acid derivatives could also have an improved anti-cancer activity [35,36,37]. For example, Chen and co-workers synthesized a series of hybrids of scopoletin and substituted cinnamic acid as antitumor agents and the derivatives showed significant enhancement of antitumor activity as compared with the parent compound scopoletin [38]. Mao et al. adopted molecular hybridization strategy to produce bergenin-cinnamic acid hybrids and the evaluation of their antitumor activity also proved that the hybrid compounds were superior to bergenin [39]. These stimulated us to design and synthesize the hybrids of sophora alkaloids and cinnamic acid as antitumor agents by molecular hybridization strategy with improved affinity and efficacy.

Herein, a series of sophora alkaloids-cinnamic acid hybrids (including matrine-cinnamic acid hybrids, sophoridine-cinnamic acid hybrids, and sophocarpine-cinnamic acid hybrids) were synthesized and their antiproliferative activity was evaluated against a panel of human cell lines using cisplatin as reference. The most promising molecule was selected for further pharmacological mechanism studies.

## 2. Results and Discussion

### 2.1. Chemistry

**Scheme 1:**
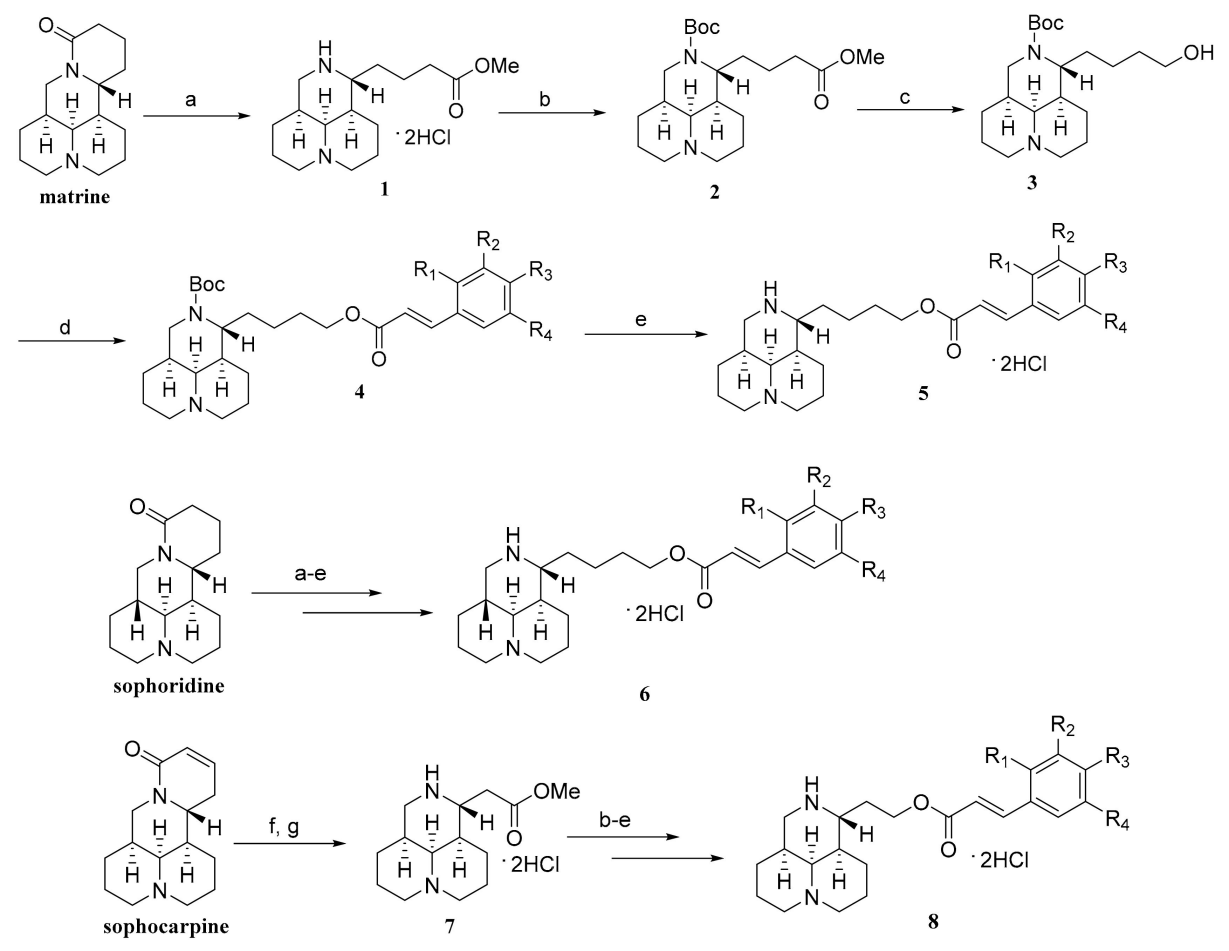
Reagents and conditions: (**a**) 2 N HCl, MeOH, reflux; (**b**) (Boc)_2_O, K_2_CO_3_, CH_2_Cl_2_; (**c**) LiAlH_4_, THF; (**d**) substituted *trans*-cinnamic acid, DMAP, EDCI, CH_2_Cl_2_; (**e**) i) CF_3_COOH, CH_2_Cl_2_, ii) HCl-Et_2_O; (**f**) KMnO_4_, 10%H_2_SO_4_; (**g**) 2 N HCl, MeOH.

The synthesis of sophora alkaloids-cinnamic derivatives is outlined in Scheme 1. Using commercially available matrine as the starting material, the intermediate methyl matrinic butyrate **1** was obtained through hydrolysis and methyl esterification via hydrochloric acid/MeOH reflux. The Boc protective group was introduced to the 12*N*-atom of **1** which produced **2** with a yield of 73%, which was used in the following reduction step by LiAlH_4_ to give **3 [40,41]**. Then, the intermediate **3** reacted with substituted trans-cinnamic acid by esterification reaction to afford **4**. Finally, deprotection of Boc group by trifluoroacetic acid produced the desired product which was slightly unstable and was further made in the form of hydrochloride salt **5**. The hydrochloride salt of sophoridine-cinnamic acid hybrid **6** was obtained from sophoridine as the starting material under the same reaction condition as matrine. For the synthesis of sophocarpine-cinnamic acid hybrids, the key intermediate **7** was acquired via the oxidation reaction from sophocarpine by KMnO_4_ and 10% H_2_SO_4_ followed by an esterification reaction. In the following procedures, the synthetic conditions were also the same as that of matrine and the hydrochloride salt of **8** was acquired. All the structures of sophora alkaloids-cinnamic acid derivatives were shown in Table 1.

### 2.2. Biological Evaluation

#### 2.2.1. Antiproliferative Activity and Structure-Activity Relationships Analysis

All of the synthesized compounds were initially evaluated for their antiproliferative activities against three cancer cell lines (HeLa, HepG2, and A549) with cisplatin as a positive reference using the MTT assay. The results were summarized in Table 2.

Compared to the parent compound matrine, most compounds exhibited promising antiproliferative activity against one or more cell lines. Among them, compounds **5b**, **5e**, **5g**, and **6d** exhibited potent broad-spectrum of activities against all the tested cell lines. It is worth noting that the above four compounds displayed good antiproliferative activity against A549 cell with IC_50_ values of 7.30 ± 0.85 μM, 6.65 ± 0.89 μM, 6.54 ± 0.86 μM, and 5.47 ± 0.88 μM, respectively, which showed 547 to 731 folds increased activity as compared to matrine.

SAR analysis was first focused on the substituents at the phenyl ring of cinnamoyl moiety of matrine-cinnamic acid hybrids. The result indicated that the introduction of the electron-withdrawing group was beneficial for antitumor activity except nitro substituent (**5h**). When electron-withdrawing chlorine atom was replaced with a weaker electron-donating methyl, the antitumor activity of **5i** slightly decreased against A549 cell line. A similar phenomenon could be found by introduction of other electron-donating groups including MeO-, -OCH_2_O-, and 3, 4, 5-trimethoxy group (**5j–5l**). By comparing the substitution position of chlorine, no significant difference was found in the antiproliferative activity, but the substitution at the para-position (**5d**) seemed to be more favorable than at the ortho- and meta- position (**5b** and **5c**) against HeLa cell line.

To investigate the (*R*)- or (*S*)-configuration of the 5-chiral center for the activity, a series of sophoridine (5*R* isomer of matrine)-cinnamic acid hybrids were prepared. The results showed that most of them displayed equal activities against HeLa and HepG2 cell lines as compared to their corresponding matrine-cinnamic acid hybrids, while some derivatives exhibited decreased activities against A549 cell line compared to their 5*S*-isomer, such as **6a**, **6g**, **6h**, and **6j**. It was noted that the nitro-substituted sophoridine-cinnamic derivative **6h** also displayed weak activity which was similar to that of **5h**, indicating that the introduction of nitro group was detrimental for activity. To investigate the influence of the chain length to activity, two sophocarpine-cinnamic acid hybrids with shortened side chains were then synthesized and both of them showed weak or no activity against the tested tumor cell lines. The result indicated that the shortening of the butyl chain to ethyl chain was detrimental for activity.

#### 2.2.2. The Morphological Analysis by Hoechst 33258 Staining

**Figure 3:**
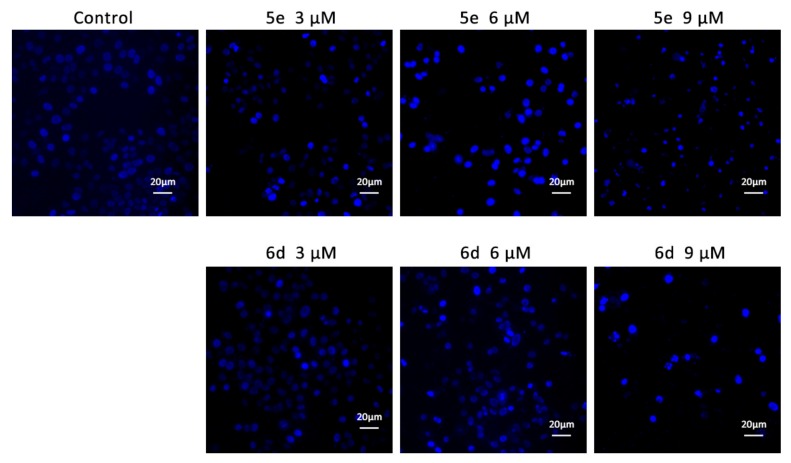
Hoechst staining for apoptosis. HepG2 Cells were treated with the indicated concentrations of **5e** and **6d** for 24 h, stained with Hoechst 33258 and examined with a fluorescent microscope.

The significant antiproliferative activity of these synthetic derivatives encouraged us to further investigate their underlying biological mechanism. As we know, the antiproliferative activity of many anti-cancer agents is closely related to the induction of apoptosis and the progress of apoptosis is often accompanied with morphological changes of chromatin. To examine whether the treatment with these compounds could lead to induce apoptosis, Hoechst 33258 staining was carried out on **5e** and **6d** treated HepG2 cells. As shown in Figure 3, the control group treated with vehicle solvent displayed uniformly light blue fluorescence, while the cells treated with **5e** and **6d** (3, 6, and 9 μM) showed sheen blue fluorescence and indicated the typical characteristics of apoptosis including nuclear fragmentation, chromatin condensation, and nuclear shrinkage. This observation revealed that apoptosis induction was involved in **5e** and **6d** treated HepG2 cells in a dose-dependent manner.

#### 2.2.3. Cell Apoptosis Assay

**Figure 4:**
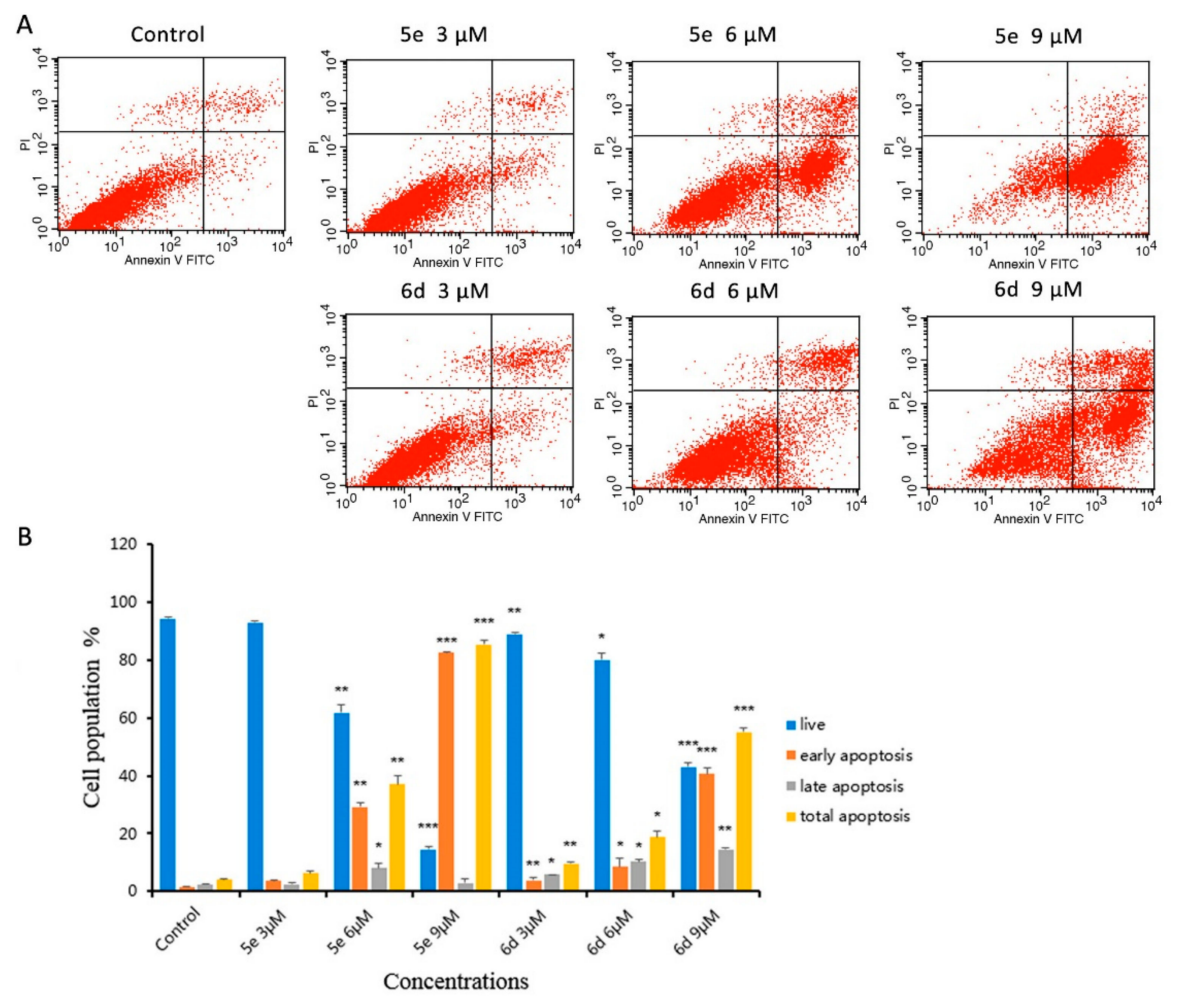
Apoptosis in HepG2 cells by treatment with **5e** and **6d**: (**A**) HepG2 cells were incubated with different concentrations of **5e** and **6d** for 24 h and the cells were stained with annexin V-FITC and PI, followed by flow cytometry analysis. (**B**) Quantitative population analysis of annexin V-FITC/PI staining, presented as mean ± SD of three individual experiments, * *p* < 0.05, ** *p* < 0.01, *** *p* < 0.001 vs control group.

We further confirmed the apoptotic-inducing effect of **5e** and **6d** on HepG2 cells through the quantitative analysis of Annexin V-FITC/propidium iodide (PI) staining by flow cytometry. As shown in Figure 4, apoptotic ratio increased accompanied with the increase of concentration of **5e** and **6d**. The treatment of **5e** gave a substantial increase in the total apoptotic ratios from 4.12% of vehicle control to 6.18% (3 μM), 37.13% (6 μM), and 85.34% (9 μM). Under the same conditions, the percentages of total apoptosis of **6d** ranged from 9.63% to 55.09%. This result proved that the compounds **5e** and **6d** induced apoptosis in a dose-dependent manner.

#### 2.2.4. Mitochondria Membrane Potential (MMP) Analysis

**Figure 5:**
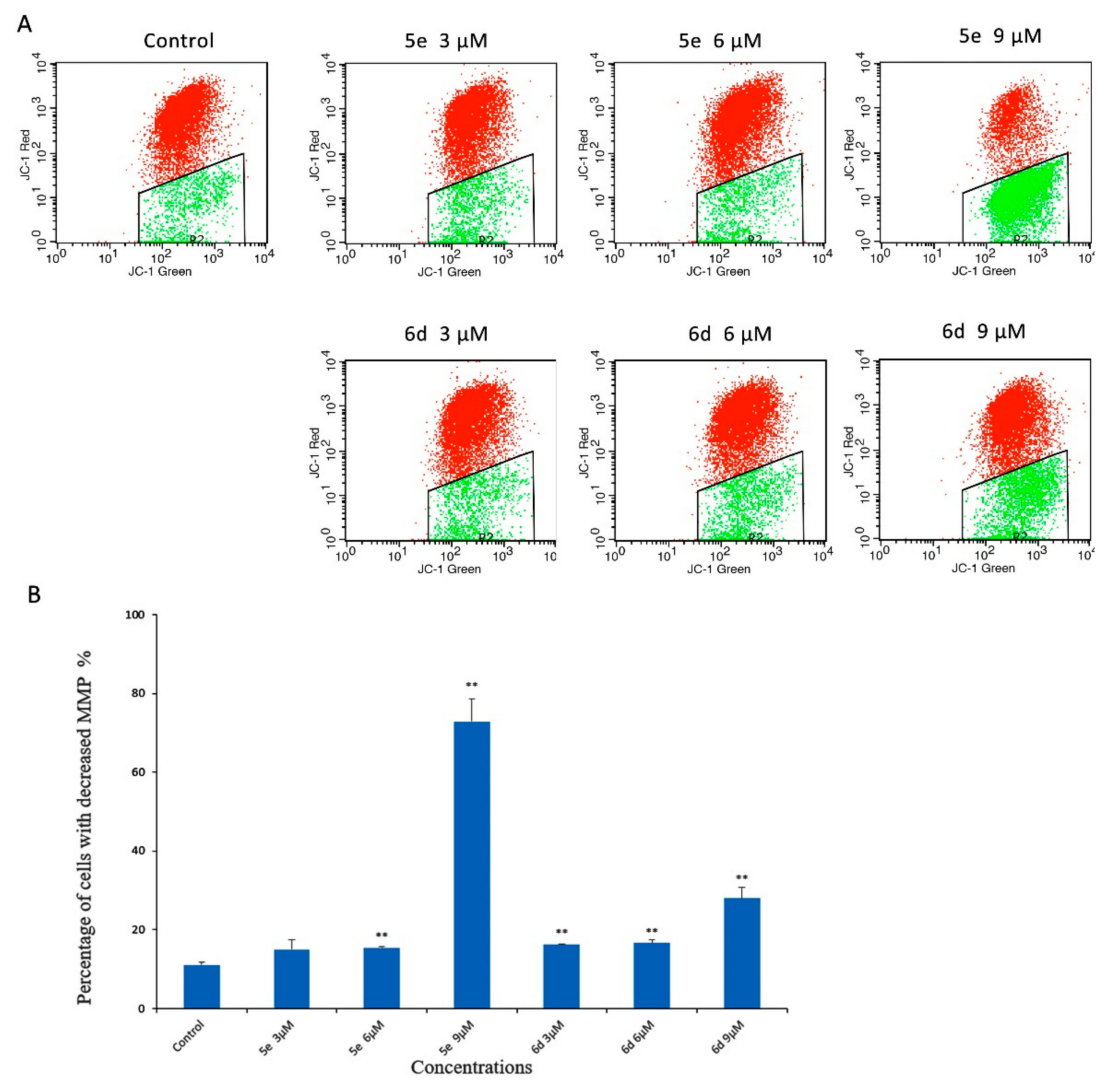
Effect of **5e** and **6d** on mitochondrial membrane potential of HepG2 cells. (**A**) Cells were treated with **5e** and **6d** for 24 h prior to staining with JC-1 and observed changes in mitochondrial membrane potential by flow cytometry. (**B**) Quantitative analysis of mitochondrial membrane potential by JC-1 staining, presented as mean ± SD of three individual experiments, * *p* < 0.05, ** *p* < 0.01 vs control group.

The apoptotic pathway of eukaryotic cells included four main pathways: The death receptor-mediated extrinsic pathway, the intrinsic mitochondrial-mediated pathway, granzyme B-mediated pathway and endoplasmic reticulum stress-mediated pathway. The disruption of mitochondrial function is considered as one of the most important apoptotic pathways, which has been recognized as an antitumor target. MMP, tested by JC-1 staining, which is an important parameter reflecting the function of mitochondria. In healthy cells with high mitochondrial membrane potential, JC-1 could spontaneously form complexes and display red fluorescence. On the other hand, in apoptotic or unhealthy cells with low MMP, JC-1 is predominantly a monomer that yields green fluorescence. The effects of **5e** and **6d** on MMP were further investigated. As shown in Figure 5, the treatment of HepG2 cells with **5e** and **6d** at different concentrations led to significantly and dose-dependently decrease of MMP. The results suggested that **5e** and **6d** could induce apoptosis through mitochondrial-mediated apoptotic pathway.

#### 2.2.5. Measurement of Intracellular Reactive Oxygen Species (ROS)

**Figure 6:**
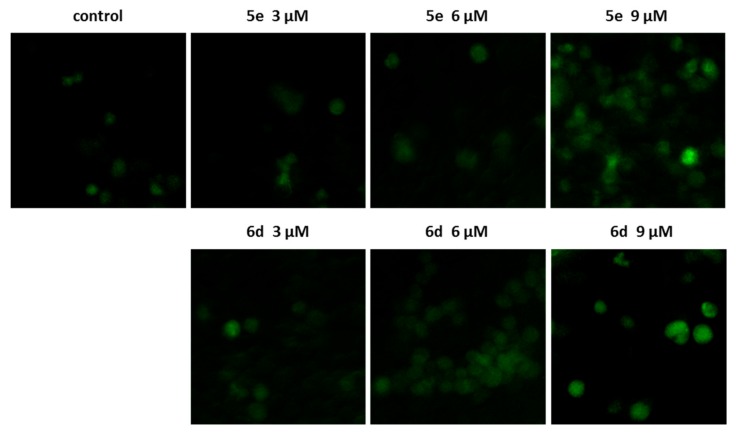
H2DCFDA staining for ROS induction. HepG2 cells were treated with indicated concentrations of **5e** and **6d** for 24 h, before staining with 10 μM H2DCFDA for 30 min.

Reactive oxygen species (ROS) are important signaling molecules associating with programmed cell death. Excessive ROS accumulation will lead to the change of mitochondrial function and the induction of apoptosis. In order to clarify whether the derivatives could increase cellular ROS levels, levels of intracellular generation of ROS upon treatment with **5e** and **6d** in HepG2 cells for 24 h were measured by 2, 7-dichlorodihydrofluorescein diacetate (H2DCFDA), a ROS-sensitive fluorescent probe. As seen in Figure 6, both **5e** and **6d** could effectively promote the production of ROS compared with the control group.

## 3. Experimental Section

NMR spectra were recorded on Bruker AV III 600 NMR spectrometer instrument (Bruker, Rheinstetten, Germany). Solvent signals (CD_3_OD: *δ*_H_ = 3.31 ppm/*δ*_C_ = 49.0 ppm) were used as reference. High resolution mass spectra (HRMS) were recorded on a Waters SYNAPT G2 HDMS (Waters, Milford, MA, USA). Reactions were monitored by thin layer chromatography on plates (GF_254_) supplied by Yantai Chemicals (Yantai, China). Silica gel column chromatography was performed using 200–300 mesh silica gel supplied by Tsingtao Haiyang Chemicals (Tsingtao, China). Unless otherwise noted, all common reagents and solvents were obtained from commercial suppliers (Beijing InnoChem Science & Technology Co., Ltd., Beijing, China) without further purification.

### 3.1. Chemistry

#### 3.1.1. Methyl Matrinate Dihydrochloride (**1**)

To a solution of matrine (5.0 g, 20.1 mmol) in MeOH (50 mL) was added 2 mol/L hydrochloric acid (30 mL) and the reaction mixture was heated to reflux for 24 h. Then the solvent was evaporated under vacuum and the residue was triturated with acetone and filtered to afford compound **1** (5.4 g, 76%).

#### 3.1.2. Methyl 12-[(Tert-Butyl)Oxycarbonyl]-Matrinate (**2**)

To a solution of compound **1** (5.0 g, 14.2 mmol) in MeOH (50 mL) was added (Boc)_2_O (4.6 g, 21.3 mmol) and anhydrous K_2_CO_3_ (5.9 g, 42.6 mmol) at room temperature. After being stirred at room temperature for 4 h, the reaction mixture was diluted with water and extracted three times with CH_2_Cl_2_. The organic layers were dried with anhydrous Na_2_SO_4_ and the solvent was removed under reduced pressure. The residue was purified by silica gel column chromatography (CH_2_Cl_2_: CH_3_OH = 40: 1, *R*_f_ = 0.3) to give compound **2** (4.8 g, 89%).

#### 3.1.3. 12-[(Tert-Butyl)Oxycarbonyl]-Matrinol (**3**)

To a solution of compound **2** (4.8 g, 12.6 mmol) in THF (30 mL) was dropwise added a solution of LiAlH_4_ (574 mg, 15.1 mmol) in THF (5 mL) at 0 °C. After being stirred at room temperature for 6 h, the reaction was quenched with acetone (5 mL) and saturated ammonium chloride solution (5 mL) was added. The mixture was stirred for 30 min and the precipitation was filtered off. The filtrate was concentrated, and the residue was dissolved in ethyl acetate, washed with water and brine, dried with anhydrous Na_2_SO_4_, and concentrated in vacuo. The residue was purified by silica gel column chromatography (CH_2_Cl_2_: CH_3_OH = 40: 1, *R*_f_ = 0.2) to give compound **3** (3.2 g, 72%).

#### 3.1.4. General Procedures for 12-[(Tert-Butyl)Oxycarbonyl]-1′-O-Substituted Cinnamoyl Matrinol (**4**)

To a solution of compound **3** (200 mg, 0.6 mmol), 4-dimethylaminopyridine (98 mg, 0.8 mmol) and 1-ethyl-3-(3-dimethyllaminopropyl) carbodiimide hydrochloride (154 mg, 0.8 mmol) in dry CH_2_Cl_2_ (5 mL) was added the corresponding cinnamic acid (0.8 mmol) at room temperature. Upon completion, the reaction mixture was diluted with water and extracted three times with CH_2_Cl_2_. The organic layers were dried with anhydrous Na_2_SO_4_ and the solvent was removed under reduced pressure. The residue was purified by silica gel column chromatography (CH_2_Cl_2_: CH_3_OH = 30: 1, *R*_f_ = 0.3) to give compound **4**.

#### 3.1.5. General Procedures for 1′-*O*-Substituted Cinnamoyl Matrinol Dihydrochloride (**5**)

To a solution of compound **4** (0.5 mmol) in CH_2_Cl_2_ (3 mL) was added trifluoroacetic acid (0.6 mL) at 0 °C and the reaction mixture was stirred at room temperature. The progress of the reaction was monitored using TLC. After completion of the reaction, the reaction was quenched with saturated NaHCO_3_ and extracted three times with CH_2_Cl_2_. The organic layers were dried with anhydrous Na_2_SO_4_ and the solvent was removed under reduced pressure. The residue was dissolved in CH_2_Cl_2_ and acidified with 2 mol/L HCl-ether (5 mL) to afford target compound **5**.

*1′-O-cinnamoyl matrinol dihydrochloride* (**5a**) white solid. Yield: 87%; mp: 156.0–157.2 °C; ^1^H-NMR (600 MHz, CD_3_OD) *δ* 7.70 (d, *J* = 16.0 Hz, 1H), 7.62–7.59 (m, 2H), 7.42–7.38 (m, 3H), 6.54 (d, *J* = 16.0 Hz, 1H), 4.28–4.23 (m, 2H), 4.11–4.04 (m, 1H), 3.84 (t, *J* = 13.6 Hz, 1H), 3.70–3.64 (m, 1H), 3.45–3.37 (m, 2H), 3.27 (dd, *J* = 13.4, 4.6 Hz, 1H), 3.09–3.00 (m, 2H), 2.52–2.44 (m, 1H), 2.26–2.20 (m, 1H), 2.12–1.91 (m, 5H), 1.90–1.74 (m, 6H), 1.73–1.53 (m, 3H); ^13^C–NMR (150 MHz, CD_3_OD) *δ* 168.6, 146.3, 135.7, 131.6, 130.1, 129.3, 118.8, 65.0, 62.7, 56.6, 56.6, 53.6, 43.8, 37.9, 32.9, 30.9, 29.5, 25.5, 24.5, 21.9, 19.6, 19.5; HRMS *m*/*z* calculated for C_24_H_35_N_2_O_2_ [M+H]^+^: 383.2699; found: 383.2691.

*1′-O-(2-chloro)cinnamoyl matrinol dihydrochloride* (**5b**) white solid. Yield: 95%. mp: 157.8–158.6 °C; ^1^H-NMR (600 MHz, CD_3_OD) *δ* 8.07 (d, *J* = 16.0 Hz, 1H), 7.79 (dd, *J* = 7.7, 1.7 Hz, 1H), 7.47 (dd, *J* = 7.9, 1.3 Hz, 1H), 7.39 (ddd, *J* = 7.9, 7.4, 1.7 Hz, 1H), 7.35 (ddd, *J* = 7.7, 7.4, 1.3 Hz, 1H), 6.58(d, *J* = 16.0 Hz, 1H), 4.28 (t, *J* = 6.4 Hz, 2H), 4.07(ddd, *J* = 12.2, 7.3, 3.4 Hz, 1H), 3.84 (t, *J* = 13.6 Hz, 1H), 3.69–3.64 (m, 1H), 3.45–3.38 (m, 2H), 3.27 (dd, *J* = 13.3, 4.6 Hz, 1H), 3.09–3.00 (m, 2H), 2.51–2.44 (m, 1H), 2.25–2.20 (m, 1H), 2.11–1.91 (m, 5H), 1.90–1.75 (m, 6H), 1.74–1.53 (m, 3H); ^13^C-NMR (150 MHz, CD_3_OD) *δ* 168.0, 141.4, 135.8, 133.6, 132.7, 131.2, 129.0, 128.7, 121.8, 65.2, 62.7, 56.6, 56.6, 53.6, 43.8, 37.9, 32.9, 30.9, 29.5, 25.5, 24.5, 21.9, 19.6, 19.5; HRMS *m*/*z* calculated for C_24_H_34_ClN_2_O_2_ [M+H]^+^: 417.2309; found: 417.2302.

*1′-O-(3-chloro)cinnamoyl matrinol dihydrochloride* (**5c**) white solid. Yield: 92%. mp: 159.6–160.5 °C; ^1^H-NMR (600 MHz, CD_3_OD) *δ* 7.68–7.63 (m, 2H), 7.55 (ddd, *J* = 6.3, 2.1, 1.8 Hz, 1H), 7.42–7.37 (m, 2H), 6.58 (d, *J* = 16.1 Hz, 1H), 4.30–4.22 (m, 2H), 4.08 (ddd, *J* = 12.2, 7.1, 3.1 Hz, 1H), 3.85 (t, *J* = 13.6 Hz, 1H), 3.69–3.65 (m, 1H), 3.45–3.38 (m, 2H), 3.28 (dd, *J* = 13.4, 4.7 Hz, 1H), 3.09–3.00 (m, 2H), 2.52–2.46 (m, 1H), 2.26–2.20 (m, 1H), 2.12–1.91 (m, 5H), 1.88–1.75 (m, 6H), 1.74–1.53 (m, 3H); ^13^C-NMR (150 MHz, CD_3_OD) *δ* 168.1, 144.5 137.8, 136.0, 131.6, 131.3, 128.9, 127.6, 120.6, 65.1, 62.7, 56.6, 56.6, 53.6, 43.8, 37.9, 32.9, 30.9, 29.5, 25.5, 24.5, 21.9, 19.6, 19.5; HRMS *m*/*z* calculated for C_24_H_34_ClN_2_O_2_ [M+H]^+^: 417.2309; found: 417.2303.

*1′-O-(4-chloro)cinnamoyl matrinol dihydrochloride* (**5d**). white solid. Yield: 91%. mp: 151.3–152.2 °C; ^1^H-NMR (600 MHz, CD_3_OD) *δ* 7.67 (d, *J* = 16.0 Hz, 1H), 7.61 (d, *J* = 8.5 Hz, 2H), 7.41 (d, *J* = 8.5 Hz, 2H), 6.55 (d, *J* = 16.0 Hz, 1H), 4.30–4.20 (m, 2H), 4.10–4.03 (m, 1H), 3.85 (t, *J* = 13.6 Hz, 1H), 3.68–3.64 (m, 1H), 3.45–3.37 (m, 2H), 3.27 (dd, *J* = 13.3, 4.4 Hz, 1H), 3.08–3.00 (m, 2H), 2.51–2.45 (m, 1H), 2.25–2.20 (m, 1H), 2.11–1.90 (m, 5H), 1.89–1.75 (m, 6H), 1.74–1.53 (m, 3H); ^13^C-NMR (150 MHz, CD_3_OD) *δ* 168.3, 144.8, 137.3, 134.5, 130.7, 130.2, 119.7, 65.1, 62.7, 56.6, 56.6, 53.6, 43.8, 38.0, 32.9, 30.9, 29.5, 25.5, 24.5, 21.9, 19.6, 19.5; HRMS *m*/*z* calculated for C_24_H_34_ClN_2_O_2_ [M+H]^+^: 417.2309; found: 417.2302.

*1′-O-(3, 4-dichloro)cinnamoyl matrinol dihydrochloride* (**5e**). white solid. Yield: 87%. mp: 152.1–153.4 °C; ^1^H-NMR (600 MHz, CD_3_OD) *δ* 7.81 (d, *J* = 1.5 Hz, 1H), 7.64 (d, *J* = 16.0 Hz, 1H), 7.58–7.54 (m, 2H), 6.60 (d, *J* = 16.0 Hz, 1H), 4.30–4.21 (m, 2H), 4.07 (ddd, *J* = 12.2, 7.1, 3.1 Hz, 1H), 3.85 (t, *J* = 13.6 Hz, 1H), 3.69–3.64 (m, 1H), 3.45–3.38 (m, 2H), 3.28 (dd, *J* = 13.4, 4.6 Hz, 1H), 3.09–3.00 (m, 2H), 2.51–2.45 (m, 1H), 2.26–2.20 (m, 1H), 2.12–1.89 (m, 5H), 1.88-1.75 (m, 6H), 1.74–1.52 (m, 3H); ^13^C-NMR (150 MHz, CD_3_OD) *δ* 168.0, 143.4, 136.3, 135.0, 134.0, 132.1, 131.0, 128.7, 121.2, 65.2, 62.7, 56.6, 56.6, 53.6, 43.8, 37.9, 32.9, 30.9, 29.5, 25.5, 24.5, 21.9, 19.6, 19.5; HRMS *m*/*z* calculated for C_24_H_33_Cl_2_N_2_O_2_ [M+H]^+^: 451.1919; found: 451.1918.

*1′-O-(3-bromo)cinnamoyl matrinol dihydrochloride* (**5f**) white solid. Yield: 98%. mp: 158.1–159.3 °C; ^1^H-NMR (600 MHz, CD_3_OD) *δ* 7.81–7.79 (m, 1H), 7.65 (d, *J* = 16.1 Hz, 1H), 7.60 (d, *J* = 7.7 Hz, 1H), 7.55 (dd, *J* = 7.9, 1.0 Hz, 1H), 7.33 (dd, *J* = 7.9, 7.7 Hz, 1H), 6.57 (d, *J* = 16.1 Hz, 1H), 4.30–4.22 (m, 2H), 4.07 (ddd, *J* = 12.2, 7.1, 3.2 Hz, 1H), 3.84 (t, *J* = 13.6 Hz, 1H), 3.68–3.64 (m, 1H), 3.45–3.38 (m, 2H), 3.27 (dd, *J* = 13.4, 4.7 Hz, 1H), 3.08-3.00 (m, 2H), 2.52–2.44 (m, 1H), 2.25–2.20 (m, 1H), 2.11–1.90 (m, 5H), 1.88–1.74 (m, 6H), 1.73–1.53 (m, 3H); ^13^C-NMR (150 MHz, CD_3_OD) *δ* 168.1, 144.5, 138.1, 134.3, 131.9, 131.8, 128.0, 124.0, 120.6, 65.1, 62.7, 56.6, 56.6, 53.6, 43.8, 37.9, 32.9, 30.9, 29.5, 25.5, 24.5, 21.9, 19.6, 19.5; HRMS *m*/*z* calculated for C_24_H_34_BrN_2_O_2_ [M+H]^+^: 461.1804; found: 461.1804.

*1′-O-(4-trifluoromethyl)cinnamoyl matrinol dihydrochloride* (**5g**) white solid. Yield: 95%. mp: 155.7–156.8 °C; ^1^H-NMR (600 MHz, CD_3_OD) *δ* 7.81 (d, *J* = 8.2 Hz, 2H), 7.75 (d, *J* = 16.1 Hz, 1H), 7.70 (d, *J* = 8.2 Hz, 2H), 6.68 (d, *J* = 16.1 Hz, 1H), 4.30–4.25 (m, 2H), 4.08 (ddd, *J* = 12.2, 7.1, 3.1 Hz, 1H), 3.85 (t, *J* = 13.6 Hz, 1H), 3.69–3.64 (m, 1H), 3.46–3.37 (m, 2H), 3.28 (dd, *J* = 13.4, 4.7 Hz, 1H), 3.09–2.98 (m, 2H), 2.52–2.45 (m, 1H), 2.26–2.20 (m, 1H), 2.12–1.91 (m, 5H), 1.90–1.75 (m, 6H), 1.74–1.53 (m, 3H); ^13^C-NMR (150 MHz, CD_3_OD) *δ* 168.0, 144.2, 139.5, 132.7 (q, *J* = 32.4 Hz), 129.7, 126.9 (q, *J* = 3.7 Hz), 125.4 (q, *J* = 269.4 Hz), 121.8, 65.2, 62.7, 56.6, 56.6, 53.7, 43.8, 38.0, 32.9, 30.9, 29.5, 25.5, 24.5, 21.9, 19.6, 19.5; HRMS *m*/*z* calculated for C_25_H_34_F_3_N_2_O_2_ [M+H]^+^: 451.2572; found: 451.2568.

*1′-O-(4-nitro)cinnamoyl matrinol dihydrochloride* (**5h**) white solid. Yield: 95%. mp: 162.1–163.4 °C; ^1^H-NMR (600 MHz, CD_3_OD) *δ* 8.26 (d, *J* = 8.8 Hz, 2H), 7.87 (d, *J* = 8.8 Hz, 2H), 7.77 (d, *J* = 16.1 Hz, 1H), 6.70 (d, *J* = 16.1 Hz, 1H), 4.32–4.24 (m, 2H), 4.08 (ddd, *J* = 12.2, 7.1, 3.1 Hz, 1H), 3.85 (t, *J* = 13.6 Hz, 1H), 3.69–3.63 (m, 1H), 3.47–3.38 (m, 2H), 3.28 (dd, *J* = 13.3, 4.6 Hz, 1H), 3.09–2.99 (m, 2H), 2.51–2.45 (m, 1H), 2.25–2.19 (m, 1H), 2.12–1.91 (m, 5H), 1.88–1.75 (m, 6H), 1.74–1.55 (m, 3H); ^13^C-NMR (150 MHz, CD_3_OD) *δ* 167.7, 150.0, 143.4, 142.0, 130.2, 125.1, 123.3, 65.3, 62.7, 56.7, 56.6, 53.7, 43.8, 38.0, 32.9, 30.9, 29.5, 25.5, 24.5, 21.9, 21.5, 19.6, 19.5; HRMS *m*/*z* calculated for C_25_H_36_N_3_O_4_ [M+H]^+^: 428.2549; found: 428.2541.

*1′-O-(4-methyl)cinnamoyl matrinol dihydrochloride* (**5i**) white solid. Yield: 94%. mp: 150.8–151.5 °C; ^1^H-NMR (600 MHz, CD_3_OD) *δ* 7.66 (d, *J* = 16.0 Hz, 1H), 7.49 (d, *J* = 8.0 Hz, 2H), 7.22 (d, *J* = 8.0 Hz, 2H), 6.47 (d, *J* = 16.0 Hz, 1H), 4.28–4.21 (m, 2H), 4.06 (ddd, *J* = 12.0, 7.0, 2.7 Hz, 1H), 3.84 (t, *J* = 13.6 Hz, 1H), 3.67-3.62 (m, 1H), 3.45–3.37 (m, 2H), 3.27 (dd, *J* = 13.3, 4.4 Hz, 1H), 3.08–2.99 (m, 2H), 2.50–2.42 (m, 1H), 2.35 (s, 3H), 2.44–2.18 (m, 1H), 2.12–1.90 (m, 5H), 1.87–1.75 (m, 6H), 1.73–1.52 (m, 3H); ^13^C-NMR (150 MHz, CD_3_OD) *δ* 168.8, 146.4, 142.3, 132.9, 130.7, 129.3, 117.7, 64.9, 62.7, 56.7, 56.6, 53.6, 43.8, 38.0, 32.9, 30.9, 29.5, 25.5, 24.5, 21.9, 21.5, 19.6, 19.5; HRMS *m*/*z* calculated for C_25_H_37_N_2_O_2_ [M+H]^+^: 397.2855; found: 397.2848.

*1′-O-(4-methoxy)cinnamoyl matrinol dihydrochloride* (**5j**) white solid. Yield: 90%. mp: 157.6–158.1 °C; ^1^H-NMR (600 MHz, CD_3_OD) *δ* 7.65 (d, *J* = 16.0 Hz, 1H), 7.55 (d, *J* = 8.7 Hz, 2H), 6.95 (d, *J* = 8.7 Hz, 2H), 6.38 (d, *J* = 16.0 Hz, 1H), 4.27–4.20 (m, 2H), 4.10–4.03 (m, 1H), 3.84 (t, *J* = 13.6 Hz, 1H), 3.82 (s, 3H), 3.68–3.62 (m, 1H), 3.46–3.38 (m, 2H), 3.27 (dd, *J* = 13.4, 4.4 Hz, 1H), 3.08–2.98 (m, 2H), 2.49–2.42 (m, 1H), 2.24–2.18 (m, 1H), 2.11–1.90 (m, 5H), 1.86–1.75 (m, 6H), 1.73–1.52 (m, 3H); ^13^C-NMR (150 MHz, CD_3_OD) *δ* 169.0, 163.2, 146.2, 131.0, 128.2, 116.0, 115.4, 64.8, 62.7, 56.7, 56.6, 55.9, 53.7, 43.8, 38.0, 32.9, 30.9, 29.6, 25.5, 24.5, 21.9, 19.6, 19.5; HRMS *m*/*z* calculated for C_25_H_37_N_2_O_3_ [M+H]^+^: 413.2804; found: 413.2802.

*1′-O-(3, 4-methylenedioxy)cinnamoyl matrinol dihydrochloride* (**5k**) white solid. Yield: 84%. mp: 152.3–153.8 °C; ^1^H-NMR (600 MHz, CD_3_OD) *δ* 7.60 (d, *J* = 15.9 Hz, 1H), 7.16 (d, *J* = 1.6 Hz, 1H), 7.09 (dd, *J* = 8.0, 1.6 Hz, 1H), 6.84 (d, *J* = 8.0 Hz, 1H), 6.36 (d, *J* = 15.9 Hz, 1H), 6.00 (s, 2H), 4.26–4.20 (m, 2H), 4.07 (ddd, *J* = 12.2, 7.2, 3.2 Hz, 1H), 3.85 (t, *J* = 13.6 Hz, 1H), 3.69–3.64 (m, 1H), 3.45–3.37 (m, 2H), 3.27 (dd, *J* = 13.3, 4.6 Hz, 1H), 3.09–3.00 (m, 2H), 2.52–2.44 (m, 1H), 2.26–2.20 (m, 1H), 2.12–1.89 (m, 5H), 1.88–1.75 (m, 6H), 1.74–1.52 (m, 3H); ^13^C-NMR (150 MHz, CD_3_OD) *δ* 168.9, 151.3, 149.9, 146.2, 130.1, 125.9, 116.6, 109.5, 107.4, 103.1, 64.9, 62.7, 56.6, 56.6, 53.6, 43.8, 37.9, 32.9, 30.9, 29.5, 25.5, 24.5, 21.9, 19.6, 19.5; HRMS *m*/*z* calculated for C_25_H_35_N_2_O_4_ [M+H]^+^: 427.2597; found: 427.2590.

*1′-O-(3, 4, 5-trimethoxy)cinnamoyl matrinol dihydrochloride* (**5l**) white solid. Yield: 85%. mp: 171.2–172.6 °C; ^1^H-NMR (600MHz, CD_3_OD) *δ* 7.64 (d, *J* = 15.9 Hz, 1H), 6.94 (s, 2H), 6.50 (d, *J* = 15.9 Hz, 1H), 4.30–4.20 (m, 2H), 4.12–4.04 (m, 1H), 3.87 (s, 6H), 3.84 (t, *J* = 13.4 Hz, 1H), 3.78 (s, 3H), 3.68–3.62 (m, 1H), 3.45–3.37 (m, 2H), 3.27 (dd, *J* = 13.4, 4.1 Hz, 1H), 3.10–2.98 (m, 2H), 2.52–2.44 (m, 1H), 2.27–2.18 (m, 1H), 2.12–1.90 (m, 5H), 1.89–1.74 (m, 6H), 1.73–1.54 (m, 3H); ^13^C-NMR (150 MHz, CD_3_OD) *δ* 168.7, 154.8, 146.4, 141.3, 131.6, 118.2, 106.7, 64.9, 62.7, 61.2, 56.8, 56.7, 56.6, 53.7, 43.8, 38.0, 32.9, 30.9, 29.5, 25.5, 24.5, 22.0, 19.6, 19.5; HRMS *m*/*z* calculated for C_27_H_41_N_2_O_5_ [M+H]^+^: 473.3015; found: 473.3011.

#### 3.1.6. 1′-*O*-Substituted Cinnamoyl Sophoridinol Dihydrochloride (**6**)

The hydrochloride salt of sophoridine-cinnamic acid hybrid **6** was obtained from sophoridine as the starting material and the synthetic route of **6** was the same as that of **5**.

*1′-O-cinnamoyl sophoridinol dihydrochloride* (**6a**) white solid. Yield: 87%. mp: 171.5–173 °C; ^1^H-NMR (600 MHz, CD_3_OD) *δ* 7.70 (d, *J* = 16.0 Hz, 1H), 7.65–7.57 (m, 2H), 7.44–7.35 (m, 3H), 6.56 (d, *J* = 16.0 Hz, 1H), 4.26 (t, *J* = 6.4 Hz, 2H), 3.83 (dd, *J* = 12.1, 4.2 Hz, 1H), 3.66–3.61 (m, 1H), 3.53 (dd, *J* = 8.9, 5.6 Hz, 1H), 3.41–3.32 (m, 2H), 3.24 (dd, *J* = 12.7, 4.7 Hz, 1H), 3.19–3.13 (m, 1H), 3.02 (t, *J* = 12.6 Hz, 1H), 2.70–2.60 (m, 1H), 2.45–2.38 (m, 1H), 2.10–1.90 (m, 5H), 1.89–1.76 (m, 5H), 1.73–1.66 (m, 1H), 1.65–1.59 (m, 1H), 1.58–1.50 (m, 1H), 1.49–1.42 (m, 1H); ^13^C-NMR (150MHz, CD_3_OD) *δ* 168.6, 146.3, 135.7, 131.6, 130.1, 129.3, 118.9, 65.1, 59.3, 58.0, 54.1, 46.4, 43.6, 35.8, 29.5, 29.1, 26.9, 26.5, 23.9, 23.8, 22.8, 18.7; HRMS *m*/*z* calculated for C_24_H_35_N_2_O_2_ [M+H]^+^: 383.2699; found: 383.2699.

*1′-O-(2-chloro)cinnamoyl sophoridinol dihydrochloride* (**6b**) white solid. Yield: 92%. mp: 143.0–143.5 °C; ^1^H-NMR (600 MHz, CD_3_OD) *δ* 8.07 (d, *J* = 16.0 Hz, 1H), 7.81 (dd, *J* = 7.7, 1.7 Hz, 1H), 7.47 (dd, *J* = 8.0, 1.1 Hz, 1H), 7.39 (ddd, *J* = 8.0, 7.4, 1.7 Hz, 1H), 7.35 (ddd, *J* = 7.7, 7.4, 1.1 Hz, 1H), 6.60 (d, *J* = 16.0 Hz, 1H), 4.28 (t, *J* = 6.4 Hz, 2H), 3.84 (dd, *J* = 12.2, 4.5 Hz, 1H), 3.63 (td, *J* = 12.9, 3.0 Hz, 1H), 3.53 (dd, *J* = 9.3, 5.5 Hz, 1H), 3.43–3.32 (m, 2H), 3.24 (dd, *J* = 13.1, 4.4 Hz, 1H), 3.19–3.13 (m, 1H), 3.03 (t, *J* = 12.5, 1H), 2.70–2.60 (m, 1H), 2.43 (dt, *J* = 13.2, 3.8 Hz, 1H), 2.10–1.92 (m, 5H), 1.89–1.78 (m, 5H), 1.73–1.67 (m, 1H), 1.66–1.59 (m, 1H), 1.58–1.51 (m, 1H), 1.50–1.42 (m, 1H); ^13^C-NMR (150 MHz, CD_3_OD) *δ* 168.1, 141.4, 135.8, 133.6, 132.7, 131.2, 129.0, 128.7, 121.8, 65.3, 59.3, 58.0, 54.1, 46.4, 43.6, 35.8, 29.4, 29.0, 26.9, 26.5, 23.8, 23.7, 22.8, 18.7; HRMS *m*/*z* calculated for C_24_H_34_ClN_2_O_2_ [M+H]^+^: 417.2309; found: 417.2313.

*1′-O-(3-chloro)cinnamoyl sophoridinol dihydrochloride* (**6c**) white solid. Yield: 89%. mp: 135.4–136.8 °C; ^1^H-NMR (600 MHz, CD_3_OD) *δ* 7.67–7.63 (m, 2H), 7.56 (ddd, *J* = 6.4, 2.4, 1.6 Hz, 1H), 7.41–7.37 (m, 2H), 6.60 (d, *J* = 16.1 Hz, 1H), 4.26 (t, *J* = 6.4 Hz, 2H), 3.83 (dd, *J* = 12.2, 4.4 Hz, 1H), 3.63 (td, *J* = 12.9, 3.0 Hz, 1H), 3.53 (dd, *J* = 9.4, 5.5 Hz, 1H), 3.42–3.32 (m, 2H), 3.24 (dd, *J* = 13.1, 4.4 Hz, 1H), 3.19–3.14 (m, 1H), 3.03 (t, *J* = 12.7 Hz, 1H), 2.70–2.61 (m, 1H), 2.44 (dt, *J* = 13.1, 3.8 Hz, 1H), 2.10–1.91 (m, 5H), 1.89–1.77 (m, 5H), 1.72-1.66 (m, 1H), 1.66–1.60 (m, 1H), 1.59–1.51 (m, 1H), 1.50–1.41 (m, 1H); ^13^C-NMR (150 MHz, CD_3_OD) *δ* 168.2, 144.5, 137.8, 136.0, 131.6, 131.2, 128.9, 127.6, 120.7, 65.3, 59.3, 58.0, 54.1, 46.4, 43.6, 35.7, 29.4, 29.1, 26.9, 26.5, 23.8, 23.8, 22.8, 18.7; HRMS *m*/*z* calculated for C_24_H_34_ClN_2_O_2_ [M+H]^+^: 417.2309; found: 417.2310.

*1′-O-(4-chloro)cinnamoyl sophoridinol dihydrochloride* (**6d**). white solid. Yield: 88%. mp: 147.5–148.8 °C; ^1^H-NMR (600 MHz, CD_3_OD) *δ* 7.67 (d, *J* = 16.0 Hz, 1H), 7.62 (d, *J* = 8.5 Hz, 2H), 7.41 (d, *J* = 8.5 Hz, 2H), 6.57 (d, *J* = 16.0 Hz, 1H), 4.26 (t, *J* = 6.4 Hz, 2H), 3.82 (dd, *J* = 12.1, 4.1 Hz, 1H), 3.62 (td, *J* = 12.7, 2.5 Hz, 1H), 3.55–3.50 (m, 1H), 3.42-3.32 (m, 2H), 3.24 (dd, *J* = 13.1, 4.1 Hz, 1H), 3.20–3.13 (m, 1H), 3.02 (t, *J* = 12.7 Hz, 1H), 2.69–2.58 (m, 1H), 2.45–2.36 (m, 1H), 2.15–1.90 (m, 5H), 1.88–1.75 (m, 5H), 1.73–1.66 (m, 1H), 1.65–1.58 (m, 1H), 1.58–1.50 (m, 1H), 1.50–1.41 (m, 1H); ^13^C-NMR (150 MHz, CD_3_OD) *δ* 168.4, 144.8, 137.3, 134.5, 130.8, 130.3, 119.7, 65.2, 59.3, 58.0, 54.1, 46.4, 43.6, 35.8, 29.5, 29.1, 26.9, 26.5, 23.9, 23.7, 22.8, 18.7; HRMS *m*/*z* calculated for C_24_H_34_ClN_2_O_2_ [M+H]^+^: 417.2309; found: 417.2310.

*1′-O-(3, 4-dichloro)cinnamoyl sophoridinol dihydrochloride* (**6e**). white solid. Yield: 72%. mp: 138.8–139.4 °C; ^1^H-NMR (600 MHz, CD_3_OD) *δ* 7.82 (d, *J* = 1.8 Hz, 1H), 7.63 (d, *J* = 16.0 Hz, 1H), 7.59–7.53 (m, 2H), 6.62 (d, *J* = 16.0 Hz, 1H), 4.26 (t, *J* = 6.4 Hz, 2H), 3.83 (dd, *J* = 12.2, 4.4 Hz, 1H), 3.63 (td, *J* = 12.9, 3.0 Hz, 1H), 3.53 (dd, *J* = 9.5, 5.5 Hz, 1H), 3.41–3.32 (m, 2H), 3.24 (dd, *J* = 13.2, 4.4 Hz, 1H), 3.19–3.14 (m, 1H), 3.03 (t, *J* = 12.5 Hz, 1H), 2.69–2.60 (m, 1H), 2.44 (dt, *J* = 12.8, 3.9 Hz, 1H), 2.10–1.92 (m, 5H), 1.90–1.76 (m, 5H), 1.72–1.67 (m, 1H), 1.66–1.59 (m, 1H), 1.58–1.51 (m, 1H), 1.50–1.41 (m, 1H); ^13^C-NMR (150 MHz, CD_3_OD) *δ* 168.0, 143.4, 136.3, 135.0, 134.0, 132.1, 131.1, 128.7, 121.3, 65.3, 59.3, 58.0, 54.1, 46.4, 43.6, 35.7, 29.4, 29.1, 26.9, 26.5, 23.9, 23.7, 22.8, 18.7; HRMS *m*/*z* calculated for C_24_H_33_Cl_2_N_2_O_2_ [M+H]^+^: 451.1919; found: 451.1927.

*1′-O-(3-bromo)cinnamoyl sophoridinol dihydrochloride* (**6f**) white solid. Yield: 79%. mp: 146.0–148.0 °C; ^1^H-NMR (600 MHz, CD_3_OD) *δ* 7.80 (s, 1H), 7.64 (d, *J* = 16.0 Hz, 1H), 7.61 (d, *J* = 7.8 Hz, 1H), 7.55 (d, *J* = 7.9 Hz, 1H), 7.33 (dd, *J* = 7.9, 7.8 Hz, 1H), 6.59 (d, *J* = 16.0 Hz, 1H), 4.26 (t, *J* = 6.4 Hz, 2H), 3.83 (dd, *J* = 12.1, 2.9 Hz, 1H), 3.63 (td, *J* = 12.8, 2.5 Hz, 1H), 3.53 (dd, *J* = 9.3, 5.5 Hz, 1H), 3.41–3.32 (m, 2H), 3.24 (dd, *J* = 13.2, 4.4 Hz, 1H), 3.20–3.14 (m, 1H), 3.03 (t, *J* = 12.6 Hz, 1H), 2.70–2.60 (m, 1H), 2.47–2.40 (m, 1H), 2.11–1.92 (m, 5H), 1.90–1.77 (m, 5H), 1.72–1.67 (m, 1H), 1.66–1.60 (m, 1H), 1.59–1.51 (m, 1H), 1.50–1.42 (m, 1H); ^13^C-NMR (150MHz, CD_3_OD) *δ* 168.2, 144.4, 138.1, 134.2, 132.0, 131.8, 128.0, 123.9, 120.7, 65.3, 59.3, 58.0, 54.1, 46.4, 43.6, 35.7, 29.4, 29.1, 26.9, 26.5, 23.8, 23.8, 22.8, 18.7; HRMS *m*/*z* calculated for C_24_H_34_BrN_2_O_2_ [M+H]^+^: 461.1804; found: 461.1813.

*1′-O-(4-trifluoromethyl)cinnamoyl sophoridinol dihydrochloride* (**6g**) white solid. Yield: 92%. mp: 131.3–132.0 °C;^1^H-NMR (600 MHz, CD_3_OD) *δ* 7.82 (d, *J* = 8.3 Hz, 2H), 7.74 (d, *J* = 16.1 Hz, 1H), 7.70 (d, *J* = 8.3 Hz, 2H), 6.71 (d, *J* = 16.1 Hz, 1H), 4.27 (t, *J* = 6.4 Hz, 2H), 3.83 (dd, *J* = 12.2, 4.4 Hz, 1H), 3.63 (td, *J* = 12.9, 3.0 Hz, 1H), 3.54 (dd, *J* = 9.4, 5.4 Hz, 1H), 3.42-3.32 (m, 2H), 3.24 (dd, *J* = 13.1, 4.4 Hz, 1H), 3.19–3.13 (m, 1H), 3.03 (t, *J* = 12.7 Hz, 1H), 2.70–2.60 (m, 1H), 2.45 (dt, *J* = 13.0, 3.8 Hz, 1H), 2.12–1.93 (m, 5H), 1.89–1.76 (m, 5H), 1.73–1.67 (m, 1H), 1.66–1.60 (m, 1H), 1.59–1.51 (m, 1H), 1.50–1.42 (m, 1H);^13^C-NMR (150MHz, CD_3_OD) *δ* 168.0, 144.2, 139.5, 132.7 (q, *J* = 32.2 Hz), 129.8, 126.9 (q, *J* = 3.5 Hz), 125.4 (q, *J* = 271.5 Hz), 121.9, 65.3, 59.3, 58.0, 54.1, 46.4, 43.6, 35.7, 29.4, 29.1, 26.9, 26.5, 23.8, 23.7, 22.8, 18.7; HRMS *m*/*z* calculated for C_25_H_34_F_3_N_2_O_2_ [M+H]^+^: 451.2572; found: 451.2576.

*1′-O-(4-nitro)cinnamoyl sophoridinol dihydrochloride* (**6h**) white solid. Yield: 92%. mp: 148.0–150.0 °C; ^1^H-NMR (600 MHz, CD_3_OD) *δ* 8.25 (d, *J* = 8.7 Hz, 2H), 7.87 (d, *J* = 8.7 Hz, 2H), 7.75 (d, *J* = 16.1 Hz, 1H), 6.75 (d, *J* = 16.1 Hz, 1H), 4.27 (t, *J* = 6.2 Hz, 2H), 3.83 (dd, *J* = 12.1, 4.1 Hz, 1H), 3.63 (td, *J* = 13.0, 3.0 Hz, 1H), 3.54 (dd, *J* = 9.2, 5.3, 1H), 3.43–3.32 (m, 2H), 3.25 (dd, *J* = 13.1, 4.3 Hz, 1H), 3.20–3.14 (m, 1H), 3.03 (t, *J* = 12.6 Hz, 1H), 2.70–2.60 (m, 1H), 2.49–2.42 (m, 1H), 2.10–1.93 (m, 5H), 1.89–1.76 (m, 5H), 1.73–1.67 (m, 1H), 1.66–1.61 (m, 1H), 1.60–1.51 (m, 1H), 1.50–1.42 (m, 1H); ^13^C-NMR (150 MHz, CD_3_OD) *δ* 167.8, 149.9, 143.3, 142.0, 130.2, 125.1, 123.4, 65.4, 59.3, 58.0, 54.1, 46.4, 43.6, 35.7, 29.4, 29.1, 26.9, 26.5, 23.8, 23.7, 22.8, 18.7; HRMS *m*/*z* calculated for C_24_H_34_N_3_O_4_ [M+H]^+^: 428.2549; found: 428.2547.

*1′-O-(4-methyl)cinnamoyl sophoridinol dihydrochloride* (**6i**) white solid. Yield: 90%. mp: 129.7–130.5 °C; ^1^H-NMR (600 MHz, CD_3_OD) *δ* 7.65 (d, *J* = 16.0 Hz, 1H), 7.50 (d, *J* = 8.2 Hz, 2H), 7.22 (d, *J* = 8.2 Hz, 2H), 6.49 (d, *J* = 16.0 Hz, 1H), 4.24 (t, *J* = 6.4 Hz, 2H), 3.83 (dd, *J* = 12.2, 4.5 Hz, 1H), 3.63 (td, *J* = 12.9, 3.0 Hz, 1H), 3.53 (dd, *J* = 9.3, 5.5, 1H), 3.42-3.31 (m, 2H), 3.24 (dd, *J* = 13.2, 4.4 Hz, 1H), 3.19–3.13 (m, 1H), 3.02 (t, *J* = 12.7 Hz, 1H), 2.70–2.60 (m, 1H), 2.47–2.40 (m, 1H), 2.35 (s, 3H), 2.10–1.92 (m, 5H), 1.88–1.76 (m, 5H), 1.72–1.66 (m, 1H), 1.66–1.59 (m, 1H), 1.59–1.51 (m, 1H), 1.50–1.42 (m, 1H); ^13^C-NMR (150 MHz, CD_3_OD) *δ* 168.8, 146.3, 142.2, 132.9, 130.7, 129.3, 117.8, 65.1, 59.3, 58.0, 54.1, 46.4, 43.6, 35.8, 29.5, 29.1, 26.9, 26.5, 23.8, 23.8, 22.8, 21.5, 18.7; HRMS *m*/*z* calculated for C_25_H_37_N_2_O_2_ [M+H]^+^: 397.2855; found: 397.2856.

*1′-O-(4-methoxy)cinnamoyl sophoridinol dihydrochloride* (**6j**) white solid. Yield: 81%. mp: 169.0–170.0 °C; ^1^H-NMR (600 MHz, CD_3_OD) *δ* 7.64 (d, *J* = 16.0 Hz, 1H), 7.56 (d, *J* = 8.8 Hz, 2H), 6.95 (d, *J* = 8.8 Hz, 2H), 6.40 (d, *J* = 16.0 Hz, 1H), 4.24 (t, *J* = 6.4 Hz, 2H), 3.85–3.80 (m, 4H), 3.63 (td, *J* = 12.9, 2.6 Hz, 1H), 3.53 (dd, *J* = 9.2, 5.5 Hz, 1H), 3.42–3.32 (m, 2H), 3.24 (dd, *J* = 13.1, 4.4 Hz, 1H), 3.19–3.13 (m, 1H), 3.02 (t, *J* = 12.7 Hz, 1H), 2.70–2.60 (m, 1H), 2.46–2.39 (m, 1H), 2.10–1.92 (m, 5H), 1.87–1.77 (m, 5H), 1.72–1.66 (m, 1H), 1.65–1.59 (m, 1H), 1.58–1.51 (m, 1H), 1.50–1.42 (m, 1H); ^13^C-NMR (150 MHz, CD_3_OD) *δ* 169.1, 163.2, 146.1, 131.0, 128.2, 116.1, 115.4, 65.0, 59.3, 58.0, 55.9, 54.1, 46.4, 43.6, 35.8, 29.5, 29.1, 26.9, 26.5, 23.8, 23.8, 22.8, 18.7; HRMS *m*/*z* calculated for C_25_H_37_N_2_O_3_ [M+H]^+^: 413.2804; found: 413.2806.

*1′-O-(3, 4-methylenedioxy)cinnamoyl sophoridinol dihydrochloride* (**6k**) white solid. Yield: 95%. mp: 142.7–143.5 °C; ^1^H-NMR (600 MHz, CD_3_OD) *δ* 7.60 (d, *J* = 15.9 Hz, 1H), 7.17 (d, *J* = 1.3 Hz, 1H), 7.09 (dd, *J* = 8.0, 1.3 Hz, 1H), 6.84 (d, *J* = 8.0 Hz, 1H), 6.38 (d, *J* = 15.9 Hz, 1H), 6.00 (s, 2H), 4.23 (t, *J* = 6.4 Hz, 2H), 3.83 (dd, *J* = 12.0, 3.8 Hz, 1H), 3.63 (td, *J* = 12.8, 2.3 Hz, 1H), 3.53 (dd, *J* = 9.1, 5.4 Hz, 1H), 3.42–3.32 (m, 2H), 3.24 (dd, *J* = 13.1, 4.2 Hz, 1H), 3.19–3.13 (m, 1H), 3.02 (t, *J* = 12.6 Hz, 1H), 2.70–2.60 (m, 1H), 2.44–2.40 (m, 1H), 2.12–1.91 (m, 5H), 1.88–1.75 (m, 5H), 1.71–1.67 (m, 1H), 1.66–1.59 (m, 1H), 1.58–1.51 (m, 1H), 1.50–1.42 (m, 1H); ^13^C-NMR (150 MHz, CD_3_OD) *δ* 168.9, 151.3, 149.9, 146.1, 130.1, 125.9, 116.7, 109.5, 107.4, 103.1, 65.0, 59.3, 58.0, 54.1, 46.4, 43.6, 35.8, 29.5, 29.1, 26.9, 26.5, 23.8, 23.8, 22.8, 18.7; HRMS *m*/*z* calculated for C_25_H_35_N_2_O_4_ [M+H]^+^: 427.2597; found: 427.2599.

#### 3.1.7. Methyl Sophocarpinate Dihydrochloride (**7**)

To a solution of sophocarpine (2.0 g, 8.1 mmol) in 10% H_2_SO_4_ (20 mL) was slowly added KMnO_4_ (1.9 g, 12.2 mmol) in an ice bath and then the reaction mixture was heated to reflux for 24 h. After completion of the reaction, the solution was cooled to room temperature and CH_3_OH (200 mL) was added. The mixture was stirred for 10 min and the precipitation was filtered off. The filtrate was concentrated, and the residue was dissolved in CH_3_OH (20 mL) and 2 mol/L hydrochloric acid (20 mL) was added. The reaction mixture was heated to reflux until TLC analysis showed the reaction was completed. After the reaction mixture was neutralized with ammonia water to pH 6–7, the solvent was evaporated under vacuum and the residue was dissolved in CH_3_OH (100 mL). The precipitation was filtered off and the filtrate was concentrated. The residue was purified by silica gel column chromatography (CH_2_Cl_2_: CH_3_OH = 30: 1, *R*_f_ = 0.2) to give **7** (1.8 g, 68%)

The hydrochloride salt of sophocarpine-cinnamic acid hybrid **8** was obtained from **7** and the following synthetic route of **8** was the same as that of **5**.

*1′-O-(2-chloro)cinnamoyl sophocarpinol dihydrochloride* (**8a**) white solid. Yield: 94%. mp: 134.1–135.2 °C; ^1^H-NMR (600 MHz, CD_3_OD) *δ* 8.10 (d, *J* = 16.0 Hz, 1H), 7.85 (dd, *J* = 7.7, 1.7 Hz, 1H), 7.46 (d, *J* = 7.9 Hz, 1H), 7.39 (ddd, *J* = 7.9, 7.5, 1.7 Hz, 1H), 7.35 (dd, *J* = 7.7, 7.5 Hz, 1H), 6.75 (d, *J* = 16.0 Hz, 1H), 4.45–4.40 (m, 2H), 4.32–4.25 (m, 1H), 3.90 (t, *J* = 13.6 Hz, 1H), 3.74–3.68 (m, 1H), 3.47–3.39 (m, 2H), 3.33 (dd, *J* = 13.4, 4.6 Hz, 1H), 3.11–3.01 (m, 2H), 2.58–2.49 (m, 1H), 2.45–2.38 (m, 1H), 2.36–2.28 (m, 1H), 2.16 (d, *J* = 14.6 Hz, 1H), 2.09–1.91 (m, 4H), 1.91–1.74 (m, 4H); ^13^C-NMR (150 MHz, CD_3_OD) *δ* 167.7, 141.7, 135.8, 133.6, 132.7, 131.2, 129.2, 128.6, 121.7, 62.6, 61.2, 56.6, 56.6, 51.8, 43.9, 38.1, 32.8, 30.6, 25.5, 24.6, 19.6, 19.4; HRMS *m*/*z* calculated for C_22_H_30_ClN_2_O_2_ [M+H]^+^: 389.1996; found: 389.1989.

*1′-O-(4-methoxy)cinnamoyl sophocarpinol dihydrochloride* (**8b**) white solid. Yield: 90%. mp: 145.4–146.5 °C; ^1^H-NMR (600 MHz, CD_3_OD) *δ* 7.76 (d, *J* = 16.0 Hz, 1H), 7.62 (d, *J* = 8.8 Hz, 2H), 6.95 (d, *J* = 8.8 Hz, 2H), 6.50 (d, *J* = 16.0 Hz, 1H), 4.42 (dt, *J* = 11.9, 5.5 Hz, 1H), 4.39–4.32 (m, 1H), 4.29 (ddd, *J* = 12.5, 8.0, 2.9 Hz, 1H), 3.91 (t, *J* = 13.7 Hz, 1H), 3.82 (s, 3H), 3.71–3.68 (m, 1H), 3.47–3.39 (m, 2H), 3.32 (dd, *J* = 13.4, 4.6 Hz, 1H), 3.11–3.00 (m, 2H), 2.57–2.50 (m, 1H), 2.44–2.36 (m, 1H), 2.35–2.28 (m, 1H), 2.14 (d, *J* = 11.5 Hz, 1H), 2.10–1.91 (m, 4H), 1.91–1.75 (m, 4H); ^13^C-NMR (150 MHz, CD_3_OD) *δ* 168.6, 163.3, 146.7, 131.2, 128.3, 115.8, 115.4, 62.6, 60.8, 56.7, 56.6, 55.9, 51.8, 43.9, 38.2, 32.8, 30.8, 25.5, 24.6, 19.6, 19.4; HRMS *m*/*z* calculated for C_23_H_33_N_2_O_3_ [M+H]^+^: 385.2491; found: 385.2490.

The NMR spectra of the compounds **5a**–**5l**, **6a**–**6k** and **8a**-**8b** are shown in the electronic Supplementary Materials.

### 3.2. Biological Evaluation

#### 3.2.1. Cell Lines and Cell Culture

The cell lines, Cervical (HeLa), Lung (A549) and Liver (HepG2) which were used in this study were procured from National Infrastructure of Cell Line Resource and were cultured in Dulbecco’s Modified Eagle Medium supplemented with 10% fetal bovine serum, 100 U/mL penicillin and 100 μg/mL streptomycin. The cells were maintained in a humidified incubator with 95% air and 5% CO_2_ at 37 °C.

#### 3.2.2. Antiproliferative Activity

The antiproliferative activity of the synthetic compounds was evaluated by the MTT assay in vitro, with cisplatin as a positive control. In brief, the cells were seeded into 96-well plates at the density of 5 × 10^3^ cells/well and were cultured overnight for adherence. The test compounds were prepared from 4 mM or 16 mM stock solution in DMSO according to preliminary screening results. Then these stocks solution were serially diluted with DMEM medium with the final DMSO concentration no greater than 0.5%. The concentration was set to 20 μM, 10 μM, 5 μM, 2.5 μM, and 1.25 μM for potent compounds and 80 μM, 40 μM, 20 μM, 10 μM, and 5 μM for less potent compounds. The cells were treated with different concentrations of test compounds for 48 h. After that, 20 μL MTT (5 mg/mL) were added to each well and the plates were further incubated for 4 h. The medium was carefully removed and the formazan crystals were dissolved in 150 μL DMSO. The absorbance at 490 nm was measured on a microplate reader (TECAN Infinite M1000, Austria). The cell viability was determined in terms of IC_50_ value.

#### 3.2.3. Hoechst 333258 Staining

HepG2 cells were seeded at a density of 2 × 10^5^ cells/well on coverslips in 6-well plates and allowed to attach overnight, the media was replaced with fresh media containing with vehicle or compounds **5e** and **6d** at a dose of 3, 6, and 9 μM, respectively and further incubated for 24 h. The cells were washed with PBS twice and stained with Hoechst 333258 (5 μg/mL) for 15 min at 37 °C. After washing the coverslips twice with PBS, the cells were visualized with a fluorescence microscopy (Zeiss, Germany) with a DAPI filter.

#### 3.2.4. Cell Apoptosis Assay

Apoptosis was detected by flow cytometry using Annexin V-FITC/Propidium Iodide (PI) double staining assay. Compounds **5e** and **6d** were chosen for cell apoptosis assay, and the concentration was set to 3 μM, 6 μM, and 9 μM by diluting the 4M stock solution. The final concentrations of DMSO were 0.075%, 0.15%, and 0.225%, respectively. HepG2 cells (2 × 10^5^ cells/well) were cultured in 6-well plates and allowed to attach overnight. After treatment with compounds **5e** and **6d** as described in Hoechst 333258 staining, the cells were harvested by trypsinization. According to manufacturer’s instructions of the kit (Miltenyi Biotec GmbH), resuspend the cells in 100 μL binding buffer and incubate the cell suspension with 10 μL FITC-Annexin V for 15 min at room temperature in the dark. Wash cells with binding buffer. Resuspend cell pellet in 500 μL binding buffer with 5 μL PI solution. Cellular fluorescence was measured by flow cytometry analysis (FACS Calibur^TM^, BD Biosciences, CA, USA).

#### 3.2.5. Mitochondrial Membrane Potential Assay

The percentage of decreased MMP of cells was measured using a JC-1 mitochondrial membrane potential detection kit (Beyotime, China) according to the manufacturer’s instructions. HepG2 cells were seeded at a density of 2 × 10^5^ cells/well in 6-well plates and incubated overnight. After treatment with compounds **5e** and **6d** as described in Hoechst 33258 staining, the cells were collected by trypsinization. Resuspend the cells in 0.5 mL cell culture medium, add 0.5 mL of freshly prepared JC-1 working solution and incubate for 20 min at 37 °C. in the dark. Then the cells were washed twice with staining buffer without JC-1 and subjected to flow cytometry (FACS Calibur^TM^, BD Biosciences, CA, USA).

#### 3.2.6. Measurement of Intracellular ROS Level

The fluorescent imaging of ROS generation was performed on HepG2 cells. HepG2 cells was seeded at 1 × 10^5^ cells/well in 6-well plate and were incubated at 37 °C under a 5% CO_2_ atmosphere for 24 h before treated with compounds **5e** and **6d** at the indicated time and dosages. The cells were then incubated with 10 μM H2DCFDA for 30 min, washed with PBS for twice and observed under a Zeiss Axio Observer 3 fluorescence microscope.

#### 3.2.7. Statistical Analysis

All data were expressed as mean ± SD from at least three independent experiments. SPSS 17.0 software was used for statistical analysis. Student’s *t*-test was performed. *p*-value < 0.05 was considered statistically significant.

## 4. Conclusions

A series of hybrids of sophora alkaloids and substituted cinnamic acids were designed, synthesized and evaluated in vitro against three human tumor cell lines. Some of the synthesized compounds displayed promising activities against all the tested cell lines. The structure-activity relationship study showed that the introduction of electron-withdrawing groups to the benzene ring was beneficial for the antitumor activity and shortening of the butyl chain of matrinol to ethyl chain was detrimental for activity. The mechanistic studies indicated that **5e** and **6d** could induce mitochondrial-mediated apoptosis in HepG2 cells. All considered, our studies suggested that these compounds may provide potential lead compounds for further development as cancer chemotherapeutics.

## Figures and Tables

**Table 1:**
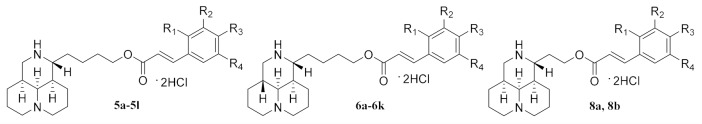
The structures of the sophora alkaloid derivatives.

Compound	Substituents			
	R_1_	R_2_	R_3_	R_4_
**5a**	H	H	H	H
**5b**	Cl	H	H	H
**5c**	H	Cl	H	H
**5d**	H	H	Cl	H
**5e**	H	Cl	Cl	H
**5f**	H	Br	H	H
**5g**	H	H	CF_3_	H
**5h**	H	H	NO_2_	H
**5i**	H	H	CH_3_	H
**5j**	H	H	OCH_3_	H
**5k**	H	OCH_2_O		H
**5l**	H	OCH_3_	OCH_3_	OCH_3_
**6a**	H	H	H	H
**6b**	Cl	H	H	H
**6c**	H	Cl	H	H
**6d**	H	H	Cl	H
**6e**	H	Cl	Cl	H
**6f**	H	Br	H	H
**6g**	H	H	CF_3_	H
**6h**	H	H	NO_2_	H
**6i**	H	H	CH_3_	H
**6j**	H	H	OCH_3_	H
**6k**	H	OCH_2_O		H
**8a**	Cl	H	H	H
**8b**	H	H	OCH_3_	H

**Table 2:** Antiproliferative activity of the synthesized derivatives against HeLa, HepG2, and A549 cells.

Compound	IC_50_ (μM) ^a,b^		
	HeLa	HepG2	A549
**5a**	10.70 ± 1.22	14.50 ± 0.37	19.73 ± 0.78
**5b**	8.15 ± 0.62	7.18 ± 1.99	7.30 ± 0.85
**5c**	9.28 ± 1.08	10.38 ± 1.38	12.17 ± 0.84
**5d**	4.67 ± 0.16	8.65 ± 0.77	12.69 ± 0.81
**5e**	5.05 ± 0.19	6.09 ± 0.54	6.65 ± 0.89
**5f**	8.12 ± 0.70	7.90 ± 0.66	12.31 ± 0.80
**5g**	5.06 ± 0.62	8.77 ± 0.96	6.54 ± 0.86
**5h**	18.52 ± 0.65	20.80 ± 2.82	22.42 ± 0.50
**5i**	6.35 ± 0.82	9.64 ± 1.92	24.40 ± 0.83
**5j**	7.54 ± 0.74	15.01 ± 1.27	19.21 ± 0.79
**5k**	9.47 ± 0.56	11.53 ± 1.21	21.50 ± 0.76
**5l**	7.76 ± 0.81	8.88 ± 0.78	12.24 ± 0.72
**6a**	9.86 ± 0.49	11.60 ± 1.29	>40
**6b**	17.06 ± 0.85	7.99 ± 0.84	14.16 ± 0.81
**6c**	11.21 ± 0.42	6.15 ± 0.56	35.88 ± 0.81
**6d**	7.93 ± 0.60	6.18 ± 0.45	5.47 ± 0.88
**6e**	6.79 ± 1.23	3.81 ± 0.24	13.48 ± 0.92
**6f**	13.98 ± 0.42	4.33 ± 0.37	14.48 ± 0.92
**6g**	7.63 ± 0.85	5.84 ± 1.18	>40
**6h**	24.61 ± 4.83	19.26 ± 2.54	>40
**6i**	6.15 ± 0.49	4.16 ± 0.29	10.63 ± 0.99
**6j**	10.02 ± 3.24	11.84 ± 0.95	>40
**6k**	13.40 ± 0.60	12.87 ± 0.78	27.27 ± 0.93
**8a**	13.54 ± 2.47	>40	>40
**8b**	13.96 ± 0.93	>40	>40
matrine	1883 ± 271	1241 ± 225	>4000
sophoridine	1532 ± 191	1035 ± 167	>4000
cisplatin	20.44 ± 6.23	39.90 ± 4.31	79.20 ± 5.60

^a^ IC_50_ values are shown as mean ± SD from the average of three replicates; ^b^ IC_50_: concentration that causes a 50% reduction of cell growth.

## Supplementary Materials

The following are available online. ^1^H-NMR and ^13^C-NMR spectra of the synthesized compounds.
