# Supplementary material for: Design and Synthesis of Molecular Hybrids of Sophora Alkaloids and Cinnamic Acids as Potential Antitumor Agents
Source: Molecules. 2020 Mar 5;25(5):1168. doi: 10.3390/molecules25051168 (PMC7179170; doi:10.3390/molecules25051168)
Supplement: Supplementary file 1 [file molecules-25-01168-s001.zip › Supporting Information.doc]

NMR Spectrum

^1^H NMR Spectrum for **5a** (CD_3_OD, 600 MHz)

^13^C NMR Spectrum for **5a** (CD_3_OD, 150 MHz)

^1^H NMR Spectrum for **5b** (CD_3_OD, 600 MHz)

^13^C NMR Spectrum for **5b** (CD_3_OD, 150 MHz)

^1^H NMR Spectrum for **5c** (CD_3_OD, 600 MHz)

^13^C NMR Spectrum for **5c** (CD_3_OD, 150 MHz)

^1^H NMR Spectrum for **5d** (CD_3_OD, 600 MHz)

^13^C NMR Spectrum for **5d** (CD_3_OD, 150 MHz)

^1^H NMR Spectrum for **5e** (CD_3_OD, 600 MHz)

^13^C NMR Spectrum for **5e** (CD_3_OD, 150 MHz)

^1^H NMR Spectrum for **5f** (CD_3_OD, 600 MHz)

^13^C NMR Spectrum for **5f** (CD_3_OD, 150 MHz)

^1^H NMR Spectrum for **5g** (CD_3_OD, 600 MHz)

^13^C NMR Spectrum for **5g** (CD_3_OD, 150 MHz)

^1^H NMR Spectrum for **5h** (CD_3_OD, 600 MHz)

^13^C NMR Spectrum for **5h** (CD_3_OD, 150 MHz)

^1^H NMR Spectrum for **5i** (CD_3_OD, 600 MHz)

^13^C NMR Spectrum for **5i** (CD_3_OD, 150 MHz)

^1^H NMR Spectrum for **5j** (CD_3_OD, 600 MHz)

^13^C NMR Spectrum for **5j** (CD_3_OD, 150 MHz)

^1^H NMR Spectrum for **5k** (CD_3_OD, 600 MHz)

^13^C NMR Spectrum for **5k** (CD_3_OD, 150 MHz)

^1^H NMR Spectrum for **5l** (CD_3_OD, 600 MHz)

^13^C NMR Spectrum for **5l** (CD_3_OD, 150 MHz)

^1^H NMR Spectrum for **6a** (CD_3_OD, 600 MHz)

^13^C NMR Spectrum for **6a** (CD_3_OD, 150 MHz)

^1^H NMR Spectrum for **6b** (CD_3_OD, 600 MHz)

^13^C NMR Spectrum for **6b** (CD_3_OD, 150 MHz)

^1^H NMR Spectrum for **6c** (CD_3_OD, 600 MHz)

^13^C NMR Spectrum for **6c** (CD_3_OD, 150 MHz)

^1^H NMR Spectrum for **6d** (CD_3_OD, 600 MHz)

^13^C NMR Spectrum for **6d** (CD_3_OD, 150 MHz)

^1^H NMR Spectrum for **6e** (CD_3_OD, 600 MHz)

^13^C NMR Spectrum for **6e** (CD_3_OD, 150 MHz)

^1^H NMR Spectrum for **6f** (CD_3_OD, 600 MHz)

^13^C NMR Spectrum for **6f** (CD_3_OD, 150 MHz)

^1^H NMR Spectrum for **6g** (CD_3_OD, 600 MHz)

^13^C NMR Spectrum for **6g** (CD_3_OD, 150 MHz)

^1^H NMR Spectrum for **6h** (CD_3_OD, 600 MHz)

^13^C NMR Spectrum for **6h** (CD_3_OD, 150 MHz)

^1^H NMR Spectrum for **6i** (CD_3_OD, 600 MHz)

^13^C NMR Spectrum for **6i** (CD_3_OD, 150 MHz)

^1^H NMR Spectrum for **6j** (CD_3_OD, 600 MHz)

^13^C NMR Spectrum for **6j** (CD_3_OD, 150 MHz)

^1^H NMR Spectrum for **6k** (CD_3_OD, 600 MHz)

^13^C NMR Spectrum for **6k** (CD_3_OD, 150 MHz)

^1^H NMR Spectrum for **8a** (CD_3_OD, 600 MHz)

^13^C NMR Spectrum for **8a** (CD_3_OD, 150 MHz)

^1^H NMR Spectrum for **8b** (CD_3_OD, 600 MHz)

^13^C NMR Spectrum for **8b** (CD_3_OD, 150 MHz)
